# The stigma of epilepsy and its effects on marital status

**DOI:** 10.1186/2193-1801-3-762

**Published:** 2014-12-23

**Authors:** Hamidreza Riasi, Ali Rajabpour Sanati, Kazem Ghaemi

**Affiliations:** Department of Neuroradiology, University of Zurich, Zurich, Switzerland; Birjand University of Medical Sciences, Birjand, Iran; F. 2, NO 16, Sepehr #8, Sepehr St., Faramarz Abbasi #25, Faramarz Abbasi Ave., Mashhad, Iran; Department of Neurosurgery, Birjand University of Medical Sciences, Birjand, Iran

**Keywords:** Epilepsy, Marital status, Stigma, Iran

## Abstract

**Background:**

No previous studies have examined marital status of patients with epilepsy and epilepsy-related factors on perceived and enacted stigmas in Iran. In the present study, marital status of patients with epilepsy (PWE’s) in Birjand city in the east of Iran was investigated.

**Methods:**

A multicenter cross-sectional study was conducted to identify factors contributing to the marital status of PWE’s in a cross-sectional study with 471 participants. Diagnosis of epilepsy in participants (374 cases) was confirmed by at least two neurologists.

**Results:**

Marriage rate of PWE’s was 27.3% (n = 102 patients) and divorce rate was 54.8% (n = 205 patients). Divorce rate in women was significantly higher than in men (62.6% vs. 46.4%; P < 0.05), and there were no significant differences between the different types of epilepsy (P > 0.05).

**Conclusions:**

The stigma of epilepsy has impacts on marital status of PWE’s. The PWE’s suffering from the enacted stigma of epilepsy are significantly more likely to get divorced in comparison with other patients.

**Electronic supplementary material:**

The online version of this article (doi:10.1186/2193-1801-3-762) contains supplementary material, which is available to authorized users.

## 1. Introduction

Epilepsy is characterized with an enduring predisposition to experience seizures, and later neurobiological, cognitive, and social consequences (Fisher et al. 2005). Epilepsy has been posed as one of the health problems in developing countries (Preux & Druet-Cabanac 2005; Birbeck et al. 2007) like Iran (Masoudnia 2009). Previous studies reported that the quality of life in patients with epilepsy (PWE) not only was related to their seizure duration and extent of seizure control but also to social factors. Insufficient knowledge about epilepsy has been associated with a negative attitude and belief towards the patients and a tendency to stigmatize this condition (Amoroso et al. 2006). In the Iranian culture, the issue of epilepsy is similar to an incurable disease, which is better to separate from and avoid persons who suffer from this disease. When a person is diagnosed with it, s/he should carry the load of burden induced by the family and culture. Thus, in Iran, the mere diagnosis of epilepsy can bring high prices for the patient. Hence, it is not unexpected in married life where one of the couples may be ignored in his/her relationship upon this diagnosis.

Epilepsy has impacts on various aspects of social life of PWE. These patients are less likely to get married and more likely to get divorced in comparison with the general population (Agarwal et al. 2006; Wada et al. 2001). This social problem can be attributed to the social stigmatization of epilepsy. Stigma is a degrading and debasing attitude of the society that discredits a person or a group because of an attribute (such as an illness, deformity, color, nationality, religion, etc. The resulting coping behavior of the affected person results in internalized stigma. This perceived or internalized stigma by the discredited individual is equally destructive whether or not actual discrimination occurs. Stigma destroys a person’s dignity, marginalizes affected individuals, violates basic human rights, markedly diminishes the chances of a stigmatized person of achieving full potential, and seriously hampers pursuit of happiness and contentment (http://www.whocanyoutell.org/what-is-stigma/2014). On the contrary, externalized stigma refers to environmental issues surrounding the person through social discussion and judgment by culture and its dominant view.

This stigmatization varies across different countries and cultures (Agarwal et al. 2006; Wada et al. 2001; Jacoby 2002; Kim et al. 2003). It is necessary to distinguish between perceived and enacted stigma. Patients with perceived stigma are ashamed of having epilepsy and fear from encountering with other people, whereas the enacted stigma refers to the actual episode of discrimination (Jacoby 2002).

Patients with epilepsy (PWE’s) usually do not inform their spouses about their illness before marriage because of their fear from its consequences and impacts on marriage negotiations (perceived stigma) (Agarwal et al. 2006; Jacoby 2002). Concealing epilepsy in order to be treated as “normal” on the part of PWE’s will be often conducive to legally legitimize reasons for the other partner to file an application for divorce later. Unfortunately, there are few studies worldwide and no studies in Iran for assessment of marital situations in PWE’s. The present study is aimed to evaluate the marital status of PWE’s in Birjand city in the east of Iran.

## 2. Participants and methods

In the present cross-sectional study, we assessed the marital status of PWE’s within the age range of 18 to 40 years between July and December 2012 in order to acquire basic data and to clarify factors that affect the marital status of PWE’s in Birjand. Our statistical population consisted of PWE’s who referred to the Vali-e-Asr and Imam Reza Hospitals in Birjand City during the study. This study was approved by Committees of Research and Medical Ethics in Birjand University of Medical Sciences (BUMS). Informed consent to participate in the study was obtained from the patients.

Epilepsy in the participants was confirmed by taking medical history and physical examination by at least two neurologists. A multicenter cross-sectional study was conducted to identify factors contributing to the marital status of PWE’s.

Due to unavailability of EEG/Video EEG at the centers where the study was conducted, diagnosing epilepsy and rule outing pseudo-seizures was based on clinical procedures.

During the study period, 471 patients referred to the hospitals. 97 patients including patients with non-epileptic convulsion or unconfirmed epilepsy, patients addicted to alcohol or any type of opium, patients with major diseases such as Cerebrovascular Accident (CVA), patients with intelligence quotient less than 70 (IQ ≤ 70), patients with irregular visits, and patients who were not willing to participate in the study were excluded (Thus, the overall exclusion rate of people in statistical analysis was 12.9%). In fact our final assessment was performed on 374 patients (179 men and 195 women).

The survey questionnaire contained 21 items/questions with 3 categories: demographic variables (gender, age, level of education), epilepsy profile (age of onset, type) and social status (marital status, concealment of epilepsy from spouse before marriage) (Additional file 1). The medical records of the participants were reviewed retrospectively at each center to characterize clinical features.

Statistical analysis was done with SPSS software (version 19.0 for Windows; SPSS Inc., Chicago, IL). The analysis included the use of t-test for independent samples, Pearson’s Chi-Square, and Fisher’s exact to evaluate the statistical significance of the differences in the survey responses. Logistic regression analysis was used in an attempt to clarify the potential contributing factors affecting the current marital status of participants while avoiding the possible influence on confounders on the results. In these regression analyses, we controlled age and gender factors to examine their impact on marital status of the participants. The level of statistical significance was set as p-value < 0.05.

## 3. Results

The demographic and clinical characteristics of the participants are summarized in Table 1.

**Table 1:** **Characteristics of participants**

	PWE’s		***df***	***T-test***	***p-*** value
	Men	Women			
**Age (years)**	(n = 179)	(n = 195)	372	14.21	0.51
Mean ± SD	27.44 ± 6.57	27.93 ± 7.76			
**Age range (years)**			372	5.3	<0.001
18-20	43 (24.0%)	65 (33.3%)			
21-30	72 (40.2%)	41 (21.1%)			
31-40	64 (35.8%)	89 (45.6%)			
**Age of seizure onset**			372	21.7	
Mean ± SD	12.78 ± 9.69	13.61 ± 9.42			0.45
**Age of seizure onset (years)**			372	8.3	0.4
≤10	92 (51.4%)	93 (47.7%)			
11-20	47 (26.3%)	49 (25.1%)			
21-30	25 (14.0%)	40 (20.5%)			
31-40	15 (8.4%)	13 (6.7%)			
**Level of education (years)**			372	22.5	0.015
Not educated (0)	40 (22.3%)	23 (11.8%)			
Elementary school (1–6)	39 (21.8%)	60 (30.8%)			
Secondary school (7–9)	24 (13.4%)	35 (17.9%)			
High school (10–12)	35 (19.6%)	44 (22.6%)			
College & university (>12)	41 (22.9%)	33 (16.9%)			
**Marital status**			372	32.4	<0.001
Single	19 (10.6%)	48 (24.6%)			
Married	77 (43.0%)	25 (12.8%)			
Divorced	83 (46.4%)	122 (62.6%)			
**Epilepsy classification** ^**a**^			372	9.3	0.732
TCE	152 (84.9%)	159 (81.5%)			
ME	13 (7.3%)	18 (9.2%)			
TE	11 (6.1%)	16 (8.2%)			
AE	3 (1.7%)	2 (1.0%)			
	**Undetermined** ^**b**^				97

PWE: Patients with epilepsy; n: number of respondents to the corresponding question; SD: standard deviation; TCE: Tonic-clonic epilepsy; ME: Myoclonic Epilepsy; TE: Temporal Epilepsy; AE: Absence Epilepsy.

^a^based on ILAE classification.

^b^Not included in the statistical analysis.

In brief, 374 PWEs were interviewed including 179 men and 195 women and there were no significant difference between the two genders in terms of mean age, educational level, mean age of seizure onset, and epilepsy classification^a^.

Women with epilepsy were less likely to get married than men with epilepsy (Table 1). The results showed that history of divorce had no significant difference between the different types of epilepsy (P = 0.188). The divorce history in women was significantly higher than in men (P = 0.0001).

Among the single participants, 53.7% replied that they remained unmarried because of epilepsy. A logistic regression analysis showed that the potential contributing factors affecting the celibate status in PWE’s were gender and age; a greater 7.8-fold probability of being single for women (P = 0.0001; adjusted for age) and a greater 5.6-fold probability for PWE’s aged 18–29 years than for those aged 30–40 years (p = 0.003; adjusted for gender) (Table 2).

**Table 2:** **The potential contributing factors affecting the status of single participants**

Factors	Single (n = 67)		Logistic regression		
	Yes (n = 36)	No (n = 31)	AOR (95% CI)	***p***-value	AF
Gender					
Men	5 (7.4%)	14 (20.9%)	1	-	
Women	31 (46.3%)	17 (25.4%)	7.78 (3.38-15.62)	<0.001	Age
Age					
30-40	17 (25.4%)	11 (16.4%)	1	-	
18-29	19 (28.3%)	20 (29.8%)	5.57 (1.93-16.09)	0.01	Gender
Age of seizure onset					
> 18	11 (16.4%)	13 (19.4%)	1	-	
≤ 18	25 (37.3%)	18 (26.9%)	2.03 (0.8-5.16)	0.153	Gender
Level of education					
> High school	3 (4.5%)	7 (10.4%)	1	-	
≤ High school	33 (49.2%)	24 (35.8%)	1.63 (0.93-2.88)	0.08	Age, Gender

n: number of respondents to the corresponding question; AOR: Adjusted Odds Ratio; CI: Confidence Interval, AF: Adjusted Factor.

The potential contributing factors affecting the status of divorce in PWE’s in this study were exactly the same as those affecting single PWE’s. The adjusted odds ratio of divorce was a greater 4.5-fold probability for women than for men (*p*-value = 0.001), and a greater 5.6-fold probability for PWE’s aged 18–29 than those aged 30–40 (p-value = 0.001). However, age of seizure onset and level of education did not affect the status of being single or divorced in PWE’s (Tables 2 and 3).

**Table 3:** **The potential contributing factors affecting the status of divorced participants**

Factors	Divorce (n = 205)		Logistic regression		
	Yes (n = 137)	No (n = 68)	AOR (95% CI)	***p***-value	AF
Gender					
Men	57 (27.8%)	26 (12.7%)	1	-	
Women	80 (39.0%)	42 (20.5%)	4.5 (2.65-7.65)	0.001	Age
Age					
30-40	63 (30.7%)	33 (16.1%)	1	-	
18-29	74 (36.0%)	35 (17.1%)	5.57 (1.93-16.09)	0.001	Gender
Age of seizure onset					
> 18	40 (19.5%)	15 (7.3%)	1	-	
≤ 18	97 (47.3%)	53 (25.8%)	2.03 (0.8-5.18)	0.14	Gender
Level of education					
> High school	22 (10.7%)	15 (7.3%)	1	-	
≤ High school	115 (56.1%)	53 (25.8%)	1.63 (0.93-2.88)	0.089	Age, Gender

n: number of respondents to the corresponding question; AOR: Adjusted Odds Ratio; CI: Confidence Interval, AF: Adjusted Factor.

47% of the patients with epilepsy who had seizure onset before marriage mentioned that they had not informed their spouses of their illness before marriage and during marriage negotiations (perceived stigma). About 22% of the participants who informed their spouses about their epilepsy disorder and all the patients who had not informed their spouses about their illness, stated that this factor had adverse effects on their marriage negotiations (P = 0.224). Some 65% of the married respondents stated that they were treated improperly by their spouses and about 78% said that their spouses requested for divorce. However, about 54% of the participants said that their spouse had a good understanding of their illness (Table 4).

**Table 4:** **Relationship among perceived and enacted stigma and divorce rate**

Questions	Divorce rate	df	p-value
Spouse made aware before marriage, n = 306		372	0.135
Yes (n = 164, 53.6%)	116(70.7%)		
No (n = 142, 46.4%)	89(62.7%)		
Disadvantages in marriage negotiation, n = 307		372	0.84
Yes (n = 145, 47.2%)	96(66.2%)		
No (n = 162, 52.8%)	109(67.3%)		
Treated inappropriately by spouse, n = 307		372	<0.001
Yes (n = 199, 64.8%)	157(78.9%)		
No (n = 108, 35.2%)	48(44.4%)		
Spouse filed for divorce, n = 307		372	<0.001
Yes (n = 239, 77.9%)	192(80.3%)		
No (n = 68, 22.1%)	13(19.1%)		
Spouse understands the illness well, n = 307		372	0.55
Yes (n = 164, 53.4%)	112(68.3%)		
No (n = 143, 46.6%)	93(65%)		

n: number of respondents to the corresponding question.

There were no significant differences in enacted stigma between PWE’s who informed their spouses about their illness and those who did not (Table 4). The divorce rate among patients with epilepsy who did not inform their spouse about epilepsy was 62.7%, which was almost similar to patients who informed their spouse about the illness (70.7%, P = 0.135). The results also showed that the divorce rate for PWE’s was significantly higher for those who were treated inappropriately by their spouses and filed a divorce application (78.9% and 93.7%, p = <0.001) (enacted stigma) (Table 4).

## 4. Discussion

Of the 374 PWE’s in the present study, 67 were single. The marriage rate among patients with epilepsy was 27.3%, which is 1.15% of that expected in the overall population of Birjand City with the same age range according to the 2012 data of the Iranian Statistical Information Service (http://www.sabteahval.ir/avej/) while the divorce rate among PWE’s was 54.8%, which is 26% of that expected in the overall population of Birjand City in the same age range (2012 Iranian Statistical Information Service data; http://www.sabteahval.ir/avej/; Table 1).

In a similar study that Agarwal et. al. (2006) conducted on 240 patients with epilepsy in India, it was found that PWE’s had a lower marriage rate than the general population and women with epilepsy had a higher divorce rate (Agarwal et al. 2006). Although attitude toward epilepsy has improved in recent years, negative public attitude toward epilepsy persists (Kim et al. 2003). Some people believe that PWE’s should not marry because their children might have epilepsy too although their seizures may be well controlled. Degree of noted negative ideas against PWE’s has been different in several countries and in different cultural situations (Kim et al. 2003; Diiorio et al. 2004; Spatt et al. 2005; Awad & Sarkhoo 2008; Njamnshi et al. 2009). In addition to the public negative attitude, the perceived stigma of epilepsy in PWE’s can be attributed to this matter (Jacoby 2002). We think that interaction between the externalized and internalized stigma of epilepsy might have an impact on marital status in PWE’s.

Previous studies have reported that seizure-related clinical characteristics of PWE’s such as seizure episodes and remission of the condition did not induce any negative attitude toward the current marital status (Wada et al. 2001; Sillanpää et al. 2004). In one study in South Korea, most of the people were against their children’s marriage with a PWE and the childbearing of women with epilepsy (Choi-Kwon et al. 2004).

A lower marriage rate in women with early onset seizure seems to be a global phenomenon (Agarwal et al. 2006). It seems that childhood-onset epilepsy might have indirect impacts on personality, psychosocial maturation, and academic achievement of an individual which can be transferred through parents’ lower expectations or peer group negative attitude (Sillanpää et al. 2004). These interpersonal stigmas which can happen in the interaction of PWE’s with others, both within and outside the family, might cause lower socioeconomic status in these patients (Sillanpää et al. 2004).

The present study revealed that higher divorce rate in PWE’s might be the result of concealing epilepsy from one’s spouse before marriage because of fear from unfair discrimination in various respects. This issue was a common behavior due to the perceived stigma of epilepsy and might be the main cause of divorce in PWE’s. The main victims of this perceived stigma behavior are women with epilepsy or PWE’s who have entered an arranged marriage followed by an undesirable result (Agarwal et al. 2006; Wada et al. 2001) which seems to be due to the general culture of the society. In fact, Iran’s social culture is such that when a woman suffers from a certain disease, she will have more conjugal problems than men.

The results of this study also showed that PWE’s who disclosed their illness before marriage might not suffer from serious social discrimination in conjugal life forever. The PWE’s suffering from the enacted stigma of epilepsy are significantly more likely to get divorced in comparison with other patients.

## 5. Conclusion

The perceived stigma of patients with epilepsy does have negative impacts on the marital status of PWE’s in Iran. We must encourage the public to remove the shadow over the epilepsy stigma and make a suitable milieu for PWE’s. Seizure control in PWE’s, early treatment, and family counseling will help to improve the marital status of those with marital conflicts.

## 6. Limitations of the study

Although this research was carefully prepared, we are still aware of its limitations and shortcomings. First of all, the research was conducted in two groups and lasted for 6 months. Six months is not enough for the researchers to observe all aspects of epilepsy in patients’ marital status. It would be better if it was done during a longer period of time. Also, in order to generalize the results to larger groups, the study should have involved a greater number of participants. Second, it would be better if the population of the control group increased to 2 or 3 times of the number of PWE’s for a better case–control study.

## Endnote

^a^PWE’s are only concerned.

## Electronic supplementary material

Additional file 1: Questionnaire.(DOCX 30 KB)

## Abbreviations

PWE: Patients with epilepsy.
