# Supplementary material for: The stigma of epilepsy and its effects on marital status
Source: Springerplus. 2014 Dec 23;3:762. doi: 10.1186/2193-1801-3-762 (PMC4320190; doi:10.1186/2193-1801-3-762)
Supplement: Supplementary file 1 — Additional file 1: Questionnaire.(DOCX 30 KB) [file 40064_2014_1512_MOESM1_ESM.docx]

**Appendix A**

Questionnaire

- **Name:**
- **Date of referral:**

1. Gender:
   - Female

- Male

1. How old are you? □□ years old
2. Age at seizure onset: □□ years old
3. What is your level of education?
   - I’m not educated
   - Elementary school
   - Guidance school
   - High school
   - College and university
4. Where are you from?
   - Birjand
   - Other city (please name it: …….…….)
5. Do you have any history of epilepsy in your first-degree family?
   - Yes (relation to you :…………..)
   - No
6. When was the last time you have a seizure?
   - Less than 2 years
   - More than 2 years
7. What is your marital status?
   - Single
     1. *If you are single, is it because of your epilepsy/seizure? Yes* □ *No* □
   - Married
     1. *Do you ever have divorced because of your epilepsy/seizure? Yes* □ *No* □
   - Divorced
     1. *If you are divorced, is it because of your epilepsy/seizure? Yes* □ *No* □
8. Did you ever conceal your epilepsy from your husband/wife? *(for only those who have been married)*
   - Yes, before marriage
   - Yes, he/she still doesn’t know
   - No
9. I have experienced disadvantages in marriage negotiation due to epilepsy;
   - Yes
   - No
10. I have been treated inappropriately by my spouse due to my epilepsy;
    - Yes
    - No
11. I have had divorce filed by my spouse due to epilepsy;
    - Yes
    - No
12. The spouse comprehends my illness;
    - Yes
    - No

**THE FOLLOWING 2 QUESTIONS MUST BE FILLED BY YOUR DOCTORS**

1. Type of epilepsy:

- Tonic-clonic □ Myoclonic □ Temporal □ Absence

*(Signature/Comment: ………………………………………………………………………………………………………………………)*
